# Mass spectrometry based high-throughput bioanalysis of low molecular weight compounds: are we ready to support personalized medicine?

**DOI:** 10.1007/s00216-021-03583-2

**Published:** 2021-08-23

**Authors:** Sophie Bravo-Veyrat, Gérard Hopfgartner

**Affiliations:** Life Sciences Mass Spectrometry, Department of Inorganic and Analytical Chemistry, University of Geneva, 24 Quai Ernest Ansermet, CH-1211 Geneva 4, Switzerland

**Keywords:** Liquid chromatography–mass spectrometry, Personalized medicine, Bioanalysis, High-throughput analysis, Low molecular weight compounds

## Abstract

Liquid chromatography coupled to mass spectrometry (LC-MS) is the gold standard in bioanalysis for the development of quantitative assays to support drug development or therapeutic drug monitoring. High-throughput and low-cost gene sequencing have enabled a paradigm shift from one treatment fits all to personalized medicine (PM). However, gene monitoring provides only partial information about the health state. The full picture requires the combination of gene monitoring with the screening of exogenous compounds, metabolites, lipids, and proteins. This critical review discusses how mass spectrometry–based technologies and approaches including separation sciences, ambient ionization, and ion mobility are/could be used to support high-throughput bioanalysis of endogenous end exogenous low molecular weight compounds. It includes also various biological sample types (from blood to expired air), and various sample preparation techniques.

**Graphical abstract:**

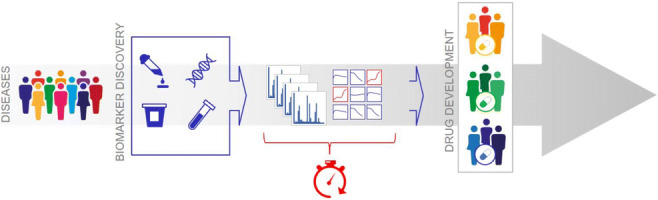

## Introduction

Completed in 2003 after 13 years, the human genome sequencing project identified approximately 21,300 genes in the human DNA sequence and has led to a better understanding of gene activity. From 2001 to 2019, the price of genome sequencing significantly dropped from 100,000,000 $ to 1000 $, providing a new opportunity for a massive genome sequencing. In 2015, former President Barack Obama announced the launching of Precision Medicine Initiative (PMI) [1]. Precision medicine considers each individual as unique with his own genetic code, but takes also into account environment and lifestyle, affecting the efficacy of a therapeutic treatment. Indeed, efficacy of commonly prescribed drugs varies from a patient to another due to complex factors (genetic mutations, sunlight exposition, smoker…) [2]. To further advance precision medicine, the PMI-cohort program, or *All of Us* research program [3], plans to gather data from 1,000,000 genomes or more, building up a big database of a wide diversity of patients. The aim of this program is to provide more targeted treatments based on highlighted genetic mutations. However, many diseases are not only explained by gene alteration but also by the accumulation of multi-environmental factors leading to complex disorders affecting genes, proteins, and metabolites.

Personalized medicine (PM) that uses information about a person to prevent, diagnose, and treat disease is mainly based on the systematic use of genetic data but also includes more and more proteomics and metabolomics data [4]. One of the first examples of PM application is the newborn bloodspot screening (NBS) which started in 1959 with Guthrie et al. [5], diagnosing phenylketonuria in newborns. In Europe, NBS began in 1964 in Poland and Belgium and was extended to other European countries from the end of the twentieth century up to 2007. In 2012, 9.5 million babies were screened for inborn errors of metabolism (IEM) [6]. 0.1% of babies were suspected of having a rare metabolism disorder who can thus benefit from medications, supplements, or diet modification to treat the disease.

To overcome such a large cohort of samples, clinical laboratories widely use tandem mass spectrometry to quantify NBS metabolites from dried blood spot (DBS) [7]. Indeed, due to the short analysis time and high sensitivity and selectivity, mass spectrometry allows the analysis of several hundreds of samples per day.

Another application of the PM is therapeutic drug monitoring (TDM) being essential in determining the correct drug taking into account specific drug metabolism and the pharmacokinetics of the patient [8]. Applied in inflammatory bowel diseases (IBD), TDM is required when a high pharmacokinetic variability interpatient is observed allowing clinical benefits to be maximized and reduce the side effects [9]. Biological samples (e.g., blood, plasma, urine, tissues) are collected from a single patient for TDM or from multiple patients in case of a large study. Endogenous metabolites are small molecules produced in the cell by metabolic reactions catalyzed by various enzymes. They can have different functions including fuel for cell growth, signaling, or stimulatory agents, and can have an inhibitory effect on enzymes. Metabolomics is the study of an individual’s metabolome, being the panel of metabolites of a particular organism. Metabolism screening is fundamental in interpreting a patient’s phenotype. Nowadays, metabolomics has become as important as the study of genes in understanding diseases.

Metabolites can be extracted from tissues, biological fluids, and cell samples. The analysis of large cohorts of biological samples is now essential to improve the statistical evaluation of typical metabolite behaviors observed with specific diseases. In case of good correlations, metabolites can be identified as biomarkers helping the diagnostic or supporting the treatment design. [10]. One of the two main challenges of PM is the storage of biological samples coming from different patients from different locations while ensuring the integrity of the samples over time. It is the role of biobanks to store samples and related information being key in investigating individual disease mechanism [11]. The second main challenge of PM is the need for analysis of wide cohorts of samples targeting specific analytes. This challenge must be taken over by fast bioanalytical methods and cheap assays.

**Fig. 1:**
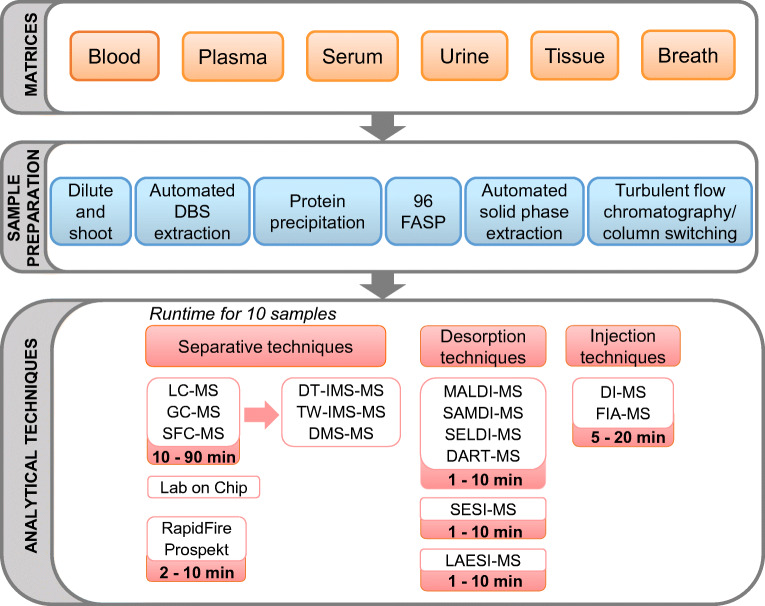
General workflow for high-throughput analysis including sample type, sample preparation, and MS based analytical techniques

In the pharmaceutical industry, high-throughput screening (HTS) started in the late 1980s mainly supporting drug discovery process. Initially, HTS was able to analyze few hundreds of compounds per week [12] and now reach up to 32,000 compounds per day, representing a maximum of 3 s per sample. The ability of an analytical technique to analyze in few seconds a sample is defined by the term “high throughput.” To be such performant, HTS needs fully automated multiplexed platforms with cost-effective and robust assays. Most of the time, fluorescence-/luminescence-based spectroscopy assays are used and measure the intensity of light emitted by cells or biochemicals. These spectroscopic methods have been recently reviewed by Dina et al. [13] where they discussed the advantage of this technique for the high-throughput monitoring of samples. Finally, mass spectrometry platforms have become the gold standard in the omics approaches to identify diseases, define treatment, or discover biomarkers in a high-throughput manner. In this review, we define here high-throughput analysis as the ability of an assay to determine several hundred samples on a daily basis or the capability of an assay to generate a valid result for a single or multiple analytes in less than a minute. This review discusses how MS-based technologies and approaches are used or could be used to support high-throughput analysis of low molecular weight compounds (e.g., pharmaceuticals, endogenous, and exogenous metabolites). Different aspects including sample type and preparation, separative techniques (mostly liquid chromatography), and multidimensional mass spectrometry are discussed and are summarized in Fig. 1.

## Sample types and sample preparation

Bioanalysis is dedicated to the quantification of drugs and their metabolites, and exogenous/endogenous molecules contained in biological matrices such as whole blood, plasma, urine, saliva, breath, or tissues. In the pharmaceutical industry, bioanalysis is used from drug discovery to post introduction of the medication. Beyond drug development, bioanalysis is applied to support clinical diagnostics, therapeutic drug monitoring in hospitals, quantification of drugs of abuse, anti-doping testing, and forensic investigation, but also for environmental monitoring pharmaceuticals in wastewater. Depending on the biological purpose, the type of samples required may be different. Classically, sample preparation is required prior to liquid chromatography coupled to mass spectrometry (LC-MS) analysis to (i) remove interferences and improve selectivity of the assay, (ii) concentrate the analyte to achieve the required limit of quantification, (iii) stabilize the analyte, or (iv) improve robustness of the assay. Often, sample preparation is optimized for a given sample volume and assay sensitivity. In PM where throughput and cost are getting critical factors, sample types, volume, and sample preparation are very important aspects to consider.

### Sample types

Indeed, each sample (venous blood, plasma, serum, or tissues) has its own specificity and needs fast and minimum handling in order to provide more reliable results [14]. In fact, it is essential to consider stability issues of the analytes in biological matrices, such as the enzymatic activity in blood or plasma which can modify the concentration of the different compounds. Thus, blood storage conditions are very important for this dynamic environment, and a storage at −80°C is usually recommended but often not practicable in hospital environments [15]. The addition of stabilization agents is often mandatory to overcome compound degradation (e.g., oxidation, hydrolysis). With the increase in sensitivity of analytical methods and in particular mass spectrometry the use of very small venous blood (e.g. 10 μl) as become possible extending the application of dried blood or plasma spot (DBS) sample collection [16]. In fact, DBS is not only a sample collection procedure which facilitates also sample storage (at ambient temperature) and transport but also an efficient sample preparation approach.

The generation of serum requires no addition of anticoagulants and is largely preferred in hospitals while most clinical studies are based on plasma samples. Several studies compared plasma and serum composition and showed high correlations between them. Yu et al. [17] reported a higher metabolite concentration in serum, but better reproducibility and sensitivity in plasma analysis. The choice of the anticoagulant (e.g., heparin, citrate, EDTA) can be critical as it can interfere with the selectivity of the analytical measurement. Urine is easy to collect and available in large quantities and is generally stored from −20 to −80°C for several months without showing any significant differences between frozen samples and fresh urine [18]. Muscles, cardiac tissues, liver, lung, placenta, arteries, and skin are the most used tissues for clinical investigation [19]. Over the years, breath is becoming as interesting as body fluid [20]. Indeed, breath carries a large amount of low molecular weight volatile organic compounds (VOCs) and its collection is as simple as breathing. Kang et al. [21] studied the stability of a breath sample trapped in a carbograph adsorbent tube and stored at −80°C, and highlighted that the endogenous compounds were stable under these conditions for 1.5 months with 94% of the VOCs remaining stable. Non-volatile compounds are also present in breath samples, exiting lungs as aerosol particles, which can be easily and selectively sampled [22]. Bruderer et al. [23] recently reviewed online analysis of exhaled breath.

### Dilute-and-shoot

Dilute-and-shoot (DnS) methodology is the simple approach and is often applied as a generic method with selective and sensitive detection such as selected reaction monitoring using triple quadrupole instruments or when large analyte coverage is desired using high-resolution mass spectrometry [24]. Typical dilution factors are between 1:1 and 1:10 with purified water or organic solvents. Sample dilution can significantly minimize matrix effects but on the cost of sensitivity [25].

### Protein precipitation

Protein precipitation (PP) is one of the most commonly used methods for sample preparation of plasma and serum samples with minimal handling. Usually, the protein precipitation procedure consists of a 1:3 (v/v) addition of an organic solvent (acetonitrile, methanol, ethanol, etc.). An alternative to organic solvents is the use of a strong acid (e.g., perchloric acid, trichloroacetic acid) or a solution containing high salt concentrations (e.g., ZnSO_4_) [26, 27]. Boernsen et al. [28] pointed out the importance of protein precipitation reaction kinetics in quantitative applications. They showed that slow reactions (made with 50% organic solvent) can avoid unwanted loss of small molecules, while fast precipitations (performed in 90% organic solvent) precipitate many small molecules leading to low recovery rates. Indeed, rapidly growing clouds of precipitated proteins disturb analyte distribution more than gradual protein precipitation. PP can easily be automated in 96 or 384 well plate format using a pipetting robot.

### Turbulent flow chromatography (TFC)

No sample preparation with direct injection of the biological fluids (e.g., plasma, urine) in a liquid chromatographic system has already been described in the early 1980s using column-switching and large particles [29, 30]. Turbulent flow chromatography (TFC) is the modern version of this approach and presents an attractive alternative to PP. Plasma can be directly injected into the chromatographic system where a column packed with large particles (25–50 μm) will only retain low molecular weight analytes [31]. Proteins, too large to be retained at such high flow rates (1.5–5.0 mL/min), are just eliminated. Recently, Song et al. [32] developed a method for large-scale quantitative analysis of compounds with a wide range of polarity, coupling column-switching, and TFC to mass spectrometry. TFC amide column was dedicated to the trapping of small apolar compounds during the loading phase. Then, the parallel back-flushing elution subsequently transmitted the compounds into the HSS T3 column for separation.

### Solid-phase extraction

Solid-phase extraction (SPE) is a largely used procedure for clean-up, extraction, and preconcentration of analytes in complex samples [33]. Various SPE materials are available in various formats, including (micro-) columns, cartridges, plates, micropipette tips, and functionalized magnetic beads (MBs) for the analysis of polar and polar compounds. Compared to liquid-liquid extraction, SPE requires less solvents and can easily be automated [34, 35]. In many cases, the SPE cartridge is used only once to avoid contamination, but affects significantly the cost of the analysis.

## Mass spectrometric detection for high-throughput analysis

### Liquid chromatography coupled to mass spectrometry

With the development of atmospheric pressure ionization (electrospray and atmospheric pressure ionization), liquid chromatography coupled to mass spectrometry has become a key technique. The sensitivity of the technique is strongly dependent on the interface design, analytical conditions, and the type of mass analyzers. A detailed analysis of 532 metabolites showed electrospray response difference (analyte analyzed under same conditions) in the range of 4–5 orders of magnitude [36]. LC-MS is a very sensitive technique and quantification limits down to 1 pg/ml in plasma with LC-MS in the selected reaction mode were already demonstrated in 1997 [37]. With conventional LC systems, analysis time in the range of 2 to 5 min can be achieved in an isocratic mode for a single analyte. Multi-analyte methods require gradient separation which result in analysis time in the range of 10–30 min and are not suitable for high-throughput analysis (HT-LC-MS) without multiplexing the number of columns. However, by optimizing various parameters such as particles size, column length, column-switching, gradient, and injection mode, an analysis time less than 22 s can be achieved [38]. One of the limited steps in HT-LC-MS remains the autosampler considering injection speed and potential crosstalk.

Core-shell, fused-core, or partially porous column stationary phases with particles down to 1.3 μm have the potential to improve analytical throughput by reducing analysis time and maintaining good chromatographic separation performance [39]. Nemkov et al. [40] reported a high-throughput 3-min method for the quantification of 35 underivatized amino acids, using a Kinetex Core-Shell XB-C18 column with 1.7 μm particle size coupled to a hybrid quadrupole Orbitrap mass spectrometer. Similar to ultra-high-performance liquid chromatography (UHPLC), supercritical fluid chromatography (SFC) enables fast analyses [41].

The use of a short column is another common way to increase the analysis throughput [42]. Indeed, with columns less than 20 mm in length, packed with small particles, fast flow rates can be applied with short (balsitic) gradients [43]. Chromatographic performance is reduced and the selectivity is mainly obtained with mass spectrometric detection. Chromatography is essential to minimize matrix effects and short column can be operated in the trap/elute mode.[42]

A commercial system such as the RapidFire (Agilent) has the advantage to pre-treat and inject the samples ultra-rapidly. This instrument, coupled to mass spectrometry, is able to perform multiple high-speed online SPE [44]. The RapidFire can provide results comparable to LC-MS but ten times faster, with runtimes typically below 15 s [45]. The Prospekt II platform, from Spark Holland, is another type of all-integrated automated sample preparation system. This online SPE device coupled to LC-MS can be used for quantitative analysis of biological fluids using a generic setup with minimal method development [46].

Improved throughput in LC-MS can also be achieved by multiplexed analyses [47]. As an example, analysis time can be reduced by overlapping injections in tandem LC configuration and column equilibration and washing can be performed during the analysis of another sample, in a parallel column. Watanabe et al. [48] developed an assay for the quantification of eight protease inhibitors using parallel ultra-high-performance liquid chromatography and mass spectrometry detection in the multiple reaction monitoring modes. With that configuration, a sample was injected every 4 min, corresponding to a 50% throughput enhancement compared to a single column experiment. There is also metabolomics multiplexed applications, such as the quantification of seven steroid hormones (cortisol, cortisone, testosterone, progesterone, corticosterone, dehydroepiandrosterone (DHEA), and androstenedione) in human hair [49].

The injection-to-injection cycle time is another parameter to optimize in order to improve the overall throughput of LC-MS experiments. The “Multiple Injections in a Single Experimental Run” (MISER) approach, which consists of sequential injections of samples at regular time intervals, allows to further decrease cycle time [38, 47, 50]. Zawatzky et al. [51] developed a high-throughput method for the enantiopurity analysis using the MISER strategy. Vistuba et al. [52] reported a decrease of analysis time by a factor of 2.6 with the use of multiple injections compared to the traditional single-injection method.

## Lab-on-a-chip techniques

Microfluidic chip technology has emerged at the end of the twentieth century and micro-technologies decrease the analysis cost while allowing a high degree of parallelization thanks to their ability to integrate microchannels. μChip-MS can be hyphenated with soft ionization techniques such as electrospray ionization (ESI) since microflows are allowed; they can also be hyphenated with matrix-assisted laser desorption/ionization (MALDI) [53]. ChipLC-MS is also used for fast sample preparation, separation, and subsequent sample analysis using MS [54].

**Fig. 2:**
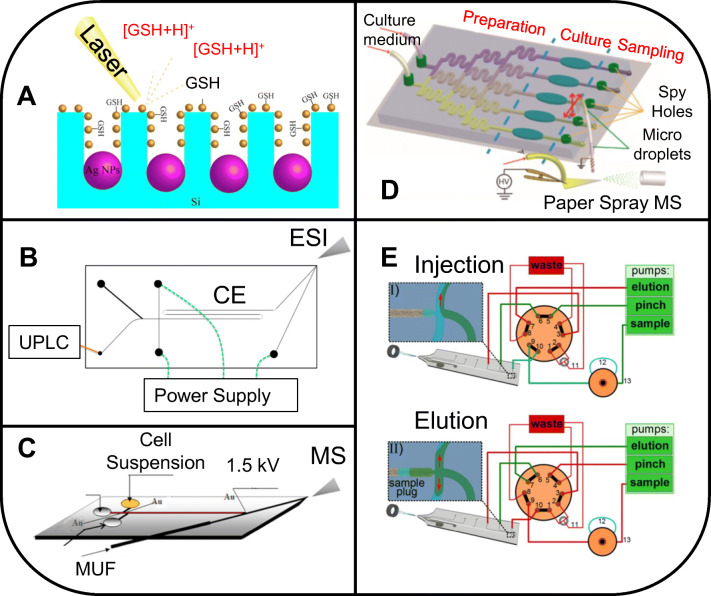
Applications using chip-MS platforms. A) Figureadapted with permission from. [63]: Gold nanoparticlesmodified porous silicon chip for SALDI-MS determination of glutathione incells. B) Hybrid capillary/microfluidic system for comprehensive online liquidchromatography-capillary electrophoresis-electrospray ionization-massspectrometry by Mellors, J. S. et al. [64],reused with permission. C) Microchip electrophoresis-mass spectrometry platformwith double cell lysis nano-electrodes for automated single cell analysis, byLi, X. et al. [65], reused with permission. D)Online Monitoring of Lactate Efflux by Multi-Channel Microfluidic Chip-MassSpectrometry for Rapid Drug Evaluation, by Liu, W. et al. [66], reused with permission. E) HPLC-MS with Glass Chips FeaturingMonolithically Integrated Electrospray Emitters of Different Geometries, byLotter, C.et al. [67], with permission

Since their introduction in 2007, microfluidic paper–based analytical devices (μPADs) are increasingly used and are cheap and easy to generate [55]. These devices can be hyphenated to ambient mass spectrometry [56]. Origami-based μPADs have emerged, allowing multiplex analysis while still being compact [57]. The development of 3D printers brings great benefits to generating μPADs in house enabling an on-demand delivery and personalized compositions and designs. μ3DPADs can be printed with cellulose powder (α-cellulose), and are able to mimic the paper texture and have the same capacity to transport the sample by capillarity [58]. Tian et al. [59] recently developed paper-based microfluidic devices for μPADs and for point-of-care testing (POCT). Lab-on-a-chip can also be used for single-cell analysis (SCA) [60]. The role of the microfluidic device is to transport, immobilize, culture, infuse with reagents, hold for observation, and retrieve single cells at high throughput [61]. Xiangtang et al. [62] developed a microchip electrophoresis–mass spectrometry (MCE-MS) platform to analyze large numbers of individual cells to determine intracellular levels of dopamine (DA) and glutamate (Glu) in PC-12 neuronal cells. Selected examples of chip-MS platforms are illustrated in Fig. 2.

## Desorption techniques and ambient ionization mass spectrometry

Introduced in 1988 [68], matrix-assisted laser desorption/ionization (MALDI) using high repetition lasers (> 1 KHz) is a high throughput–compatible method resulting in analysis times typically less than a few seconds mostly dedicated to peptides and protein anlyses. Due to interferences from the matrices and the limited resolution of TOF instruments, MALDI was unpopular for low molecular weight compound analysis (LMWC) and though to be a poor-quantitative method. However, these limitations can be overcome in MS/MS mode on quadrupole of QqTOF or with high-resolution TOF instruments using high repetition lasers (> 1 KHz), and MALDI remains an attractive approach for high-throughput analysis of LMWC [69, 70]. While MALDI is generally performed under vacuum requiring dedicated instrumentation, ambient ionization requires no matrix and allows direct analysis of samples in the open atmosphere of the laboratory and can easily be interfaced to any type of instrument [71]. Since the introduction of desorption electrospray ionization (DESI) and direct analysis in real time (DART), many different ambient ionization techniques have been reported [72] for analyte screening or quantification. As an example, Hsieh et al. [73] reported a targeted quantification analysis of endogenous cholesterol from human serum by DART-MS. In this study, only few hundred nanoliters of serum were directly collected on dried blood spots (DBS), and analyzed without any sample preparation, within a minute.

**Fig. 3:**
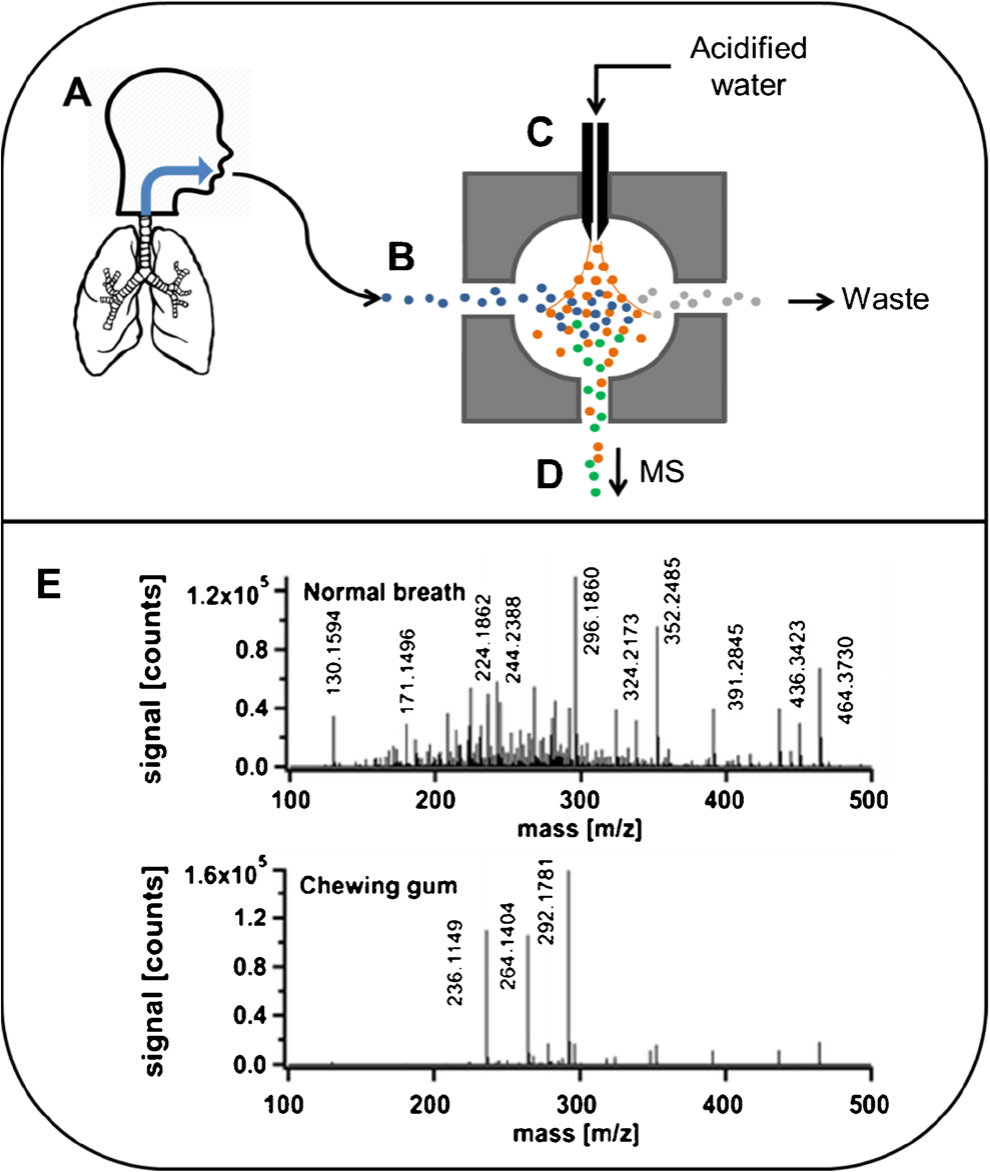
SESI workflow. (A) Exhaled breath. (B) Vaporized sample. (C) SESI ionization chamber. (D) Ionized analytes going through the mass spectrometer. (E) Example of differential breath analysis from Berchtold [75], reused with permission. On top, normal breath, and on bottom, breath spectrum after eating a chewing gum

Secondary electrospray ionization (SESI) is an additional ionization technique compatible with high-throughput analysis. This approach, based on a classical electrospray ionization source, uses a pure solvent electrospray to deliver charges into the gas-phase or liquid-sample molecules [74] (Fig. 3). Its high-throughput capability lies in the fact that there is limited or no sample preparation. Used 15 years ago for illicit drug detection and in the field of explosive analysis [76], SESI is now more focused on human skin [77] or bacterial-specific volatile metabolites [78]. SESI is also widely used for the analysis of VOCs in breath [79], as illustrated in Fig. 3.

An additional promising technique is the laser diode thermal desorption (LDTD) sample introduction source coupled with atmospheric pressure chemical ionization (APCI). LDTD-APCI sources desorb molecules from dried samples and ionize them in the corona discharge region by proton transfer [80]. Sample throughput is enhanced due to the use of multi-well plates (96- to 386-well plates). Some studies report a 50-fold analysis time reduction compared to traditional LC [81]. High-throughput applications of LDTD-APCI analysis are widespread, such as the analysis of antibiotic residues (quinolone, chemotherapeutics, sulfonamides, lincosamides, and macrolides) [82], the quantitation of cyclosporine A in whole blood for TDM [83], analysis of metformin, and sitagliptin from DBS samples in support to drug discovery [84]. In each of these applications, a very short analysis time is always reported.

In the same ambient ionization techniques category, laser ablation electrospray ionization (LAESI) is a mix between MALDI and SESI. An advantage of this technique is that, unlike MALDI, it is compatible with samples containing water. Therefore, LAESI enables less or no sample preparation. Often used in imaging (LAESI-MSI) it allows peptides and metabolites spatial resolution in tissues, in *in-vivo* living plants [85]. LAESI leads to a total analysis time of only a few seconds [75]. Another application regarding the pharmaceutical area was done by Li et al. [86] which studied the microorganism-antibiotic interactions. Desorption and ambiant ionization techniques are generally not coupled with separations science but enable rapid analyses for batch processed sampled like in mass spectrometry imaging.

## Ion mobility spectrometry

**Fig. 4:**
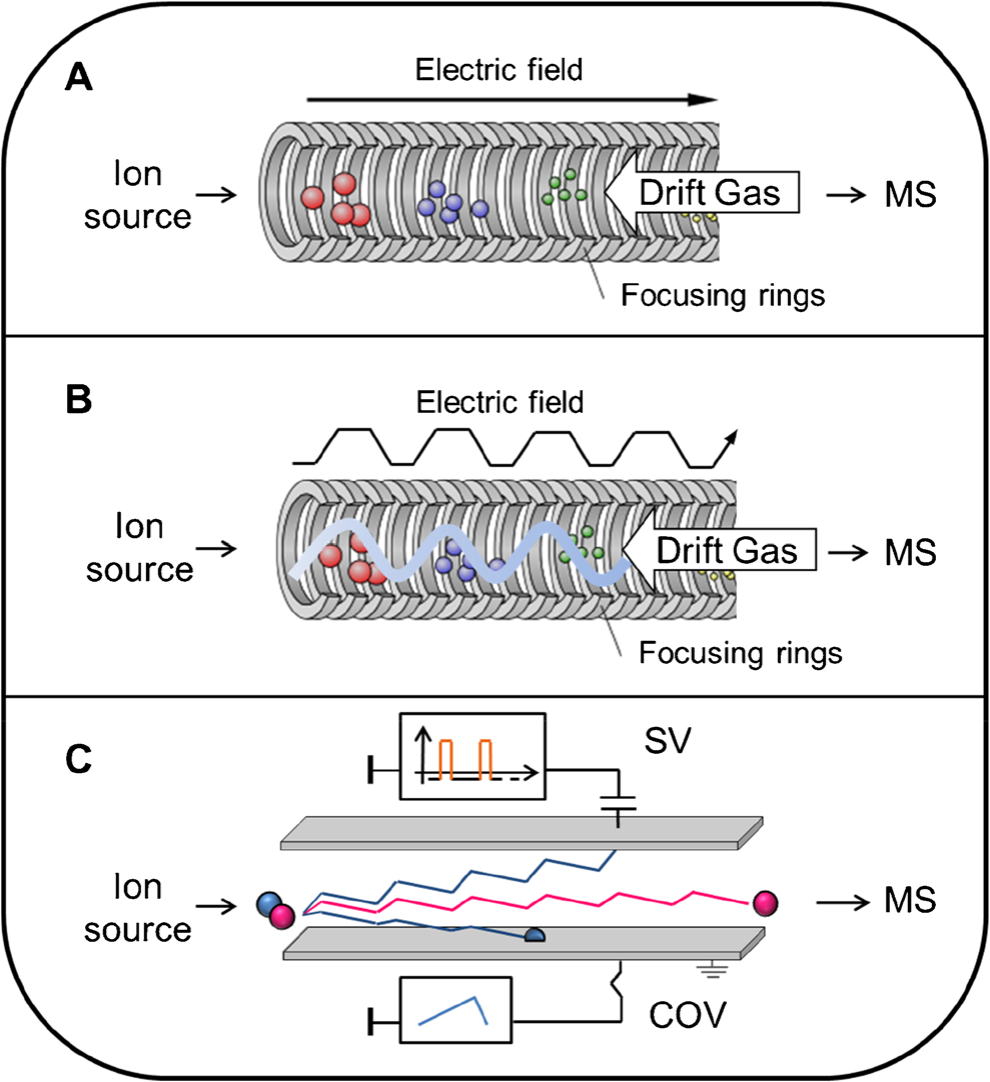
Separation principle of three different techniques of ion mobility. (A) Drift time ion mobility spectrometry. (B) Traveling-wave ion mobility spectrometry. (C) Differential mobility spectrometry

Ion mobility spectrometry (IMS) is a gas-phase electrophoretic technique, also referred as plasma chromatograph, that allows charged analytes to be separated on the basis of their mass, charge, and collision cross section (CCS) [87]. IMS was initially widely used by the military (mine detection) and for security purposes in order to fight drug trafficking and terrorist attacks. Ports and airports started to use IMS stand-alone devices, equipped with an ion collector (i.e., Faraday plate). The application of IMS has greatly been enhanced by the coupling to electrospray ionization mass spectrometers (ESI-IMS-MS) and became more used by (bio)analytical laboratories in particular for the separation of isomeric analytes or for enhanced sensitivity [88]. Various forms of IMS have been reported including drift time ion mobility (DTIMS), traveling-wave ion mobility spectrometry (TWIMS), and differential ion mobility (DMS). DTIMS is characterized by a homogeneous and continuous electric field along the drift tube in the presence of neutral gas molecules (helium or nitrogen) (see Fig. 4A). Analytes are separated by their drift time in the range of μs.

The traveling-wave ion mobility spectrometry (TWIMS) separates ions in an inhomogeneous electric field along the drift tube in the presence of neutral gas molecules. Along with the guide, the ring electrodes provide a traveling voltage wave on which the ions can be separated based on their own mobility (Fig. 4B). High-field asymmetric waveform ion mobility spectrometer (FAIMS) or differential mobility spectrometry (DMS) [89] separates ions in gas phase at atmospheric pressure, based on their difference of mobility under low and high electric fields (Fig. 4C). DMS is also used with the addition of modifiers (e.g., isopropanol, acetonitrile; toluene) and allows modifying the resolution and the selectivity of a separation. DTIMS and TWIMS are generally coupled to TOF instruments and offer the possibility to determine CCS. DMS and FAIMS are mostly applied as selectivity filters can be coupled to TOF, orbitrap, or triple quadrupole ion trap mass spectrometers. IMS-MS is gaining importance and additional forms have been reported such as trapped IMS (TIMS) [90] or cyclic IMS [91] mostly for enhanced resolution. Combined with LC, IMS can be used as an additional separation dimension but it offers also interesting possibilities for the development of high-throughput LC-IMS-MS assays [42, 43].

### Flow injection, infusion, and open-port probe mass spectrometry

Flow injection analysis (FIA) consists of the injection of a low sample volume with a LC system without a chromatographic column, and all analytes are ionized and detected by the MS instrument without prior separation. Therefore, the throughput is greatly improved, with a typical run time of less than 60 s [92]. As an example, FIA-MS applications were reported in metabolomics such as amino acid quantification, acylcarnitines, and succinylacetone from dried blood spots (DBS) by FIA-ESI-MS/MS [93]. As for LC-MS, one of the major challenges in FIA-MS is autosampler carry-over.

Direct infusion (DI) is an additional injection mode, referring to the continuous ionization of a static sample. In DI mass spectrometry (DIMS), the sample is loaded in a syringe pump which delivers it to the mass spectrometer at a constant low flow rate [94]. This technique enables the detection of multiple analytes but suffers from significant matrix effects and limited analyte dynamic range. The process can be automated using nanolectrospray infusion from a silicon chip coupled to tandem mass spectrometry for the direct analysis of drugs [95].

Open-probe sampling interface (OPSI) is a promising alternative to FIA and DIMS [96]. OPSI allows the native sample introduction directly in solvent flow from the interface of the vertically aligned, co-axial probe tube. Also known as an open-port probe (OPP), the system has been developed in order to introduce solid or liquid samples, sometimes highly concentrated and complex, to any type of atmospheric pressure ionization sources of a mass spectrometer. Ultra-high throughput can be achieved using an acoustic dispensing of individual nanoliter-volume sample droplets from microtiter plate wells into the OPSI [97] and is compatible with high-throughput workflows. Compared to flow injection analysis, OPSI in the overspill mode with a customized autosampler, equipped with disposable pipette tips, enables direct quantitative analysis of drugs in urine and plasma, with minimized carry-over and reduced matrix effects [98].

## Conclusions and perspectives

Initiated in the 1990s, Human Genome Project (HGP) was the starting point of a new way to consider medicine practice. Human DNA sequencing allowed making the link between unhealthy state and gene alterations. However, gene mutations are not the only root cause identified for a disease; indeed, multi-environmental factors have been found to play a crucial role in health. Thus, personalized medicine also considers the study of metabolites and the analysis of wide cohorts of samples to better understand the mode of action of a disease. This knowledge supports early diagnostics and treatments adapted to individuals. The screening of a large number of samples requires easy sample collection modes, and breath, saliva, urine, and capillary blood are good examples of sample types targeted by PM. However, the complexity of these biological samples is high and the use of MS is key for high-throughout analysis discriminating compounds based on their mass, where other spectroscopic methods can fail due to lack of resolution. As mass spectrometry measures mass-to-charge or mass-to-shape of charged molecules, it can be considered as a universal detector for bioanalysis that will play a major role in PM. To benefit from MS-based approaches, the technique and applications need to be shaped for the specific PM need with regard to the limit of quantification, sample throughput, cost of analysis, and data management.

Being recently commercialized, all-in-one MS platforms are attempting to overcome the lack of standardization and integrated system in clinical laboratories [99]. The Cascadion (ThermoFisher Scientific) is one example of a fully automated liquid chromatography–tandem mass spectrometry platform. Shimadzu also proposes the CLAM-2000, performing sample preparation at high throughput with LC-MS analysis.

In many cases, speed and throughput matter, and the cost per analysis is also an important factor, and currently these platforms may be too expensive considering the analysis of billions of samples may be challenging. If MS is now essential to PM, the instruments must be simplified to be handled by non-expert users to extend their use in medicine also at patient beds. An effort should also be done to make the instruments more affordable and less cumbersome. The cost of a triple quadrupole instrument is mainly driven by its sensitivity, but providers need to answer the need for speed and widespread use of MS instruments accompanying PM. One could also consider portable MS platforms available in point-of-care even for self-testing. In such point-of-care, quality control, cost optimization, reporting, archiving, and implementation of new assays could be easily implemented to support diagnostics; this is not the case with a single-use test available in pharmacies.

Beyond instrumentation, metadata generated by PM analytics rises new interrogations and worries. If bioinformatics supports the analytical instrumentation development, software must be developed to be compatible with the large amount of data collected. Indeed, high-throughput acquisition needs to be supported by high-throughput data processing, itself supported by powerful machine learning to reduce human intervention and, thus, saves time. PM appears to be promising in terms of treatment and prediction, but is also accompanied by fear from patients and professionals, related to ethical considerations. Indeed, health data security is still deemed insufficient and outdated. Despite some advances in privacy in recent years, there is still a lack of law standardization, resulting in large variations depending on patient residence place. In a bad scenario, insurance companies could misuse patient’s medical data and adjust their price accordingly.
